# Effect of Voltage and Deposition Time on Surface Morphology, Mechanical Performance, and Corrosion Resistance of Chitosan–Nanohydroxyapatite Coatings

**DOI:** 10.3390/ma19112397

**Published:** 2026-06-04

**Authors:** Klaudia Malisz-Rudzińska, Joanna Sypniewska, Marlena Grodzicka, Aleksandra Mirowska, Aleksandra Mielewczyk-Gryń, Beata Świeczko-Żurek, Alina Sionkowska

**Affiliations:** 1 Institute of Manufacturing and Materials Technology, Faculty of Mechanical Engineering and Ship Technology, Gdańsk University of Technology, Gabriela Narutowicza 11/12, 80-229 Gdansk, Poland; joanna.sypniewska@pg.edu.pl (J.S.); aleksandra.mirowska@pg.edu.pl (A.M.); beata.swieczko-zurek@pg.edu.pl (B.Ś.-Ż.); 2 Faculty of Chemistry, Nicolaus Copernicus University, Gagarina 7, 87-100 Torun, Poland; m.grodzicka@doktorant.umk.pl; 3 Institute of Nanotechnology and Materials Engineering, Advanced Materials Centre, Faculty of Applied Physics and Mathematics, Gdańsk University of Technology, Gabriela Narutowicza 11/12, 80-229 Gdansk, Poland; aleksandra.mielewczyk-gryn@pg.edu.pl

**Keywords:** chitosan, nanohydroxyapatite, nanocomposite coatings, three-point bending test, mechanical studies, corrosion test

## Abstract

**Highlights:**

- Chitosan–nanohydroxyapatite composite coatings were successfully deposited via electrophoretic deposition, demonstrating uniform coverage on both titanium alloy and stainless steel substrates.
- All coatings were continuous, free of cracks, and exhibited hydrophilic surface properties.
- Coatings deposited at a voltage of 20 V showed increased corrosion resistance while reducing hardness and stiffness—this confirms the influence of deposition voltage on the protective and mechanical properties of the coating.
- Deposition parameters strongly affected the coatings’ structural integrity, mechanical response under load, and failure behavior, highlighting the critical role of process control in optimizing CS/nHAp coatings for biomedical applications.

**Abstract:**

This study investigates the damage behavior and surface integrity of chitosan–nanohydroxyapatite (CS/nHAp) composite coatings, along with their corrosion resistance and wettability, which directly affect their biological performance in vivo. The coatings were deposited on Ti13Zr13Nb and stainless steel using electrophoretic deposition (EPD) at various voltages and deposition times. Surface topography, morphology, composition, and roughness were characterized using microscopic techniques, while wettability, corrosion resistance, and mechanical properties were assessed. Three-point bending tests were performed to determine coating behavior under mechanical deformation. Hydrophilic, homogeneous CS/nHAp coatings were successfully deposited without visible cracks on the surface. Coatings deposited at 10 V exhibited higher corrosion potentials compared to uncoated titanium alloy. Mechanical testing showed that coatings deposited at 10 V were significantly harder than those deposited at 20 V. The CS/nHAp20_5 coating exhibited moderate hardness (0.33 ± 0.06 GPa), the lowest Young’s modulus (12.7 ± 1.4 GPa), increased flexibility, and good adhesion, without delamination during bending tests. These results demonstrate that by modifying deposition parameters, it is possible to adjust the mechanical and protective properties of CS/nHAp coatings for potential application of the developed coating in vascular stents.

## 1. Introduction

Titanium and its alloys are widely used as biomaterials due to their excellent bio-compatibility and corrosion resistance in the aggressive environment of the human body. Among them, the Ti13Nb13Zr alloy exhibits one of the lowest elastic moduli of currently used titanium biomaterials (79 GPa), which is closer to that of human bone. In comparison, the elastic modulus of the Ti6Al4V alloy is approximately 140 GPa. Surface modification methods such as polishing, electrochemical oxidation, electrophoretic deposition, anodizing, ion implantation, and nitriding are widely used to improve the mechanical, biological, and physicochemical properties of titanium alloys. In addition, coatings such as hydroxyapatite, chitosan, metal oxides, or carbon-based layers can further improve surface hardness and durability [1]. An interesting topic is also nanotubular oxide layers, which not only increase the bioactivity of titanium implants but also promote the nucleation and growth of hydroxyapatite coatings [2].

Hydroxyapatite (HAp) is the mineral form of calcium apatite, most commonly represented by the formula Ca_10_(PO_4_)_6_(OH)_2_, which means that the unit cell of the crystal consists of two molecules. Hydroxyapatite is the main structural component of bones and tooth enamel. For this reason, it has found wide applications in orthopedics and dentistry. It is also used in other fields, such as environmental sciences, where it can be applied for the remediation of soil and water contaminated with heavy metals, due to its ability to easily bind with metal ions [3]. In medicine, it is often used as a filler to replace amputated bone or as a coating to support osseointegration on implants. Due to these properties, many modern bone implants are coated with hydroxyapatite. Additionally, it protects tooth enamel from acid erosion and has regenerative and anti-discoloration properties. Another interesting application is the use of hydroxyapatite as a semi-permanent filler in skin rejuvenation treatments for wrinkles and texture irregularities. As a source of phosphate and calcium ions that are slowly released into skin cells, it has even been studied for its anti-aging properties and as a sunscreen agent (a stable suspension of particles smaller than 100 nm activated with elements such as Zn, Mn, or Mg) [4]. Additionally, calcium phosphate (CaP) coatings are widely applied to magnesium-based materials to reduce their initial degradation rate. This is attributed to the fact that hydroxyapatite exhibits the lowest Gibbs free energy in physiological fluids among CaP compounds, and its chemical composition governs the degradation behavior of CaP coatings [5].

Chitosan (CS) is a natural polysaccharide polymer obtained through the deacetylation of chitin [6,7,8]. It can be prepared in various forms, such as powder, film, fiber, gel, nanoparticles, and porous structures [6]. It offers many advantages including antibacterial activity, biocompatibility, biodegradability, non-toxicity, and it is not expected to have any negative impact on surrounding tissues [6,9]. In addition, chitosan (CS) exhibits anticancer, hemostatic, and cholesterol-lowering properties. It also supports wound healing and osteogenesis. The degree of deacetylation and molecular weight can influence the properties of chitosan such as its biocompatibility, cell adhesion and proliferation, or anticoagulant activity [8,10]. However, chitosan itself has limitations in terms of mechanical properties and stability in physiological environments, which restrict its direct use as a coating for implants [7]. Additionally, a disadvantage is that chitosan is unstable in a neutral pH environment and easily absorbs water by swelling, which causes rapid drug release. The problem of too rapid degradation of coatings in physiological fluid at neutral pH can be addressed by adding Eudragit E 100, a cationic copolymer based on dimethylaminoethyl methacrylate, butyl methacrylate, and methyl methacrylate in a 2:1:1 ratio [11]. There may also be issues with the hemocompatibility of pure chitosan, but this can be improved through chemical modifications or by combining the biopolymer with compounds that exhibit complementary properties [10].

Hydroxyapatite–chitosan coatings are produced using various methods, including electrophoretic deposition from alcoholic suspensions onto metallic substrates [12,13,14]. Electrophoretic deposition is an attractive technique for producing thin films and coatings from organic, inorganic, and composite materials for biomedical applications. It involves the movement of charged particles toward an electrode under the influence of an electric field, resulting in the deposition of particles onto its surface. The advantages of this method include high purity of the deposited material, rapid deposition rate, and the ability to deposit uniform coatings on substrates with complex shapes [15]. Both chitosan and hydroxyapatite are widely used in medicine. The combination of these two compounds in composite form can contribute to improved biocompatibility and enhanced material properties. Ł. Pawłowski et al. [16] deposited composite coatings composed of chitosan, nanohydroxyapatite, and silver nanoparticles on grade 2 titanium using the electrophoretic deposition method. The effect of deposition parameters on the coating’s microstructure, adhesion, corrosion resistance, wettability, bioactivity, and silver release was investigated. Bioactive and corrosion-resistant CS/nHAp/AgNP coatings were successfully obtained, with their properties depending on the EPD parameters [16]. However, H. Kim et al. [17] developed chitosan composite scaffolds with high strength and controlled pore structure through the homogeneous dispersion of nanosized hydroxyapatite particles. The nHAp powders were dispersed in an alginate solution with a pH above 10, which served as a dispersing agent. As the nHAp content increased up to 70 wt.%, an improvement in the mechanical properties was observed, including compressive strength and elastic modulus [17]. H. Hartatiek et al. [18] also studied chitosan–hydroxyapatite-based composites in the context of mechanical testing. It was found that acidic pH conditions significantly increase the hardness of the composite [18].

This paper presents studies of composite coatings based on chitosan and hydroxyapatite, which were co-deposited using electrophoresis at various voltages and deposition times. The resulting coatings were subjected to a series of tests including analysis of surface morphology, wettability, corrosion resistance, and mechanical properties, to determine the effect of deposition parameters on the composite’s properties. The study was conducted with consideration of the potential application of the developed coating in vascular stents. Therefore, the coatings were analyzed not only on flat cylindrical samples made of a titanium alloy, but also on stainless steel wires, which better represent the geometry of the stent components. The obtained material properties were comparable for both substrate types. To further assess the mechanical integrity and adhesion of the coatings, the wires were subjected to controlled strain tests, which allowed for the assessment of the coating’s durability under mechanical stress. The study was conducted on a titanium alloy due to the growing interest in beta alloys for stent applications. Ti-Nb, Ti-Ta, Ti-Zr-Nb, Ti-Nb-Mo alloys, etc. [19,20,21] are presented as alternatives to classic materials such as stainless steel 316L, Co-Cr, Pt-Cr, or Ni-Ti, whose corrosion products cause allergic reactions. It should be emphasized that no studies describing this comparative approach for chitosan–hydroxyapatite composite coatings have been found in the available literature.

## 2. Materials and Methods

### 2.1. Preparation of the Suspension

The electrolyte was prepared in two steps. First, a suspension of nanohydroxyapatite (nHAp) powder (average grain size 20 nm, 99% purity, MK Nano, Missisauga, ON, Canada) in 99.8% ethanol was prepared according to the method described in [22]. Then, 15 mL of distilled water, 1 mL of acetic acid (99.5%), and 0.05 g of chitosan (high molecular weight, 310,000–375,000 Da; Sigma Aldrich, St. Louis, MO, USA) were added to 84 mL of the nHAp suspension (0.14 g of nHAp in 99.8% ethanol). The resulting suspension was stirred for 24 h using a magnetic stirrer and then subjected to ultrasonic treatment for 15 min immediately before deposition. The electrolyte composition for the preparation of the composite coatings was developed based on literature data [12,14,15].

### 2.2. Sample Preparation

Ti13Zr13Nb samples with a diameter of 20 mm and a height of 3 mm were used. All samples were prepared following a previously established protocol [23,24]. The surfaces were polished using SiC abrasive papers up to grit #2500. In the next step, the samples were ultrasonically cleaned in isopropanol and distilled water for 5 min each, followed by drying with compressed air.

Additionally, stainless steel wires with a diameter of 1 mm and a length of 50 mm were prepared. The surfaces were also mechanically polished using SiC abrasive papers up to grit #2500. Subsequently, the wires were ultrasonically cleaned in isopropanol and distilled water for 5 min each, followed by drying with compressed air.

### 2.3. Coating Deposition

Composite coatings were deposited on metal substrates using the electrophoretic deposition method. The titanium alloy served as the cathode, while a platinum electrode was used as the anode. The electrodes were positioned parallel to each other at a distance of 10 mm and connected to a DC power supply. EPD was performed at voltages of 10 V and 20 V for deposition times of 2 and 5 min (the sample names, depending on the applied deposition parameters, are presented in Table 1). Process parameters were determined based on literature data [12,14,15]. The process was conducted at room temperature. After deposition, the coatings were air-dried at room temperature. For coatings deposited on stainless steel wires, the wire was positioned in the center of a platinum electrode to ensure uniform coating of the entire surface. These samples were dried vertically at room temperature.

### 2.4. Surface Examinations

The topography of titanium alloy samples was studied using a Scanning Electron Microscope in secondary electrons mode (SEM, FEI Company Quanta FEG250, Hillsboro, OR, USA). Chemical composition analysis was performed using energy-dispersive X-ray spectroscopy (EDX) and Fourier-transform infrared (FTIR) for the selected samples. EDX system (Oxford Instruments, Abingdon, UK) was integrated with a scanning electron microscope (TESCAN, Brno, Czech Republic). FTIR spectra in Attenuated Total Reflectance mode (Perkin Elmer Frontier, Waltham, MA, USA) were examined at a resolution of 2 cm^−1^ in the range of 500–4000 cm^−1^.

The topography of coatings deposited on the stainless steel (SS 316L) wires was studied using a Scanning Electron Microscope (TESCAN, Brno, Czech Republic). The surface of the wires was imaged before and after deformation to assess the effect of bending on the behavior of the composite coating. Imaging was carried out at the location of bending.

The roughness of the surface was examined using a 3D optical profilometer with the confocal microscopy technique (Sensofar S Neox, Sensofar Metrology, Terrassa, Spain) with the use of Nikon-EPI 20× objective (Nikon Corporation, Tokyo, Japan). SensoSCAN S Neox 7.7 software was used to analyze the results.

The coating thickness was measured (*n* = 10) using a DualScope FMP100 coating thickness gauge (SN150001281; Helmut Fischer GmbH, Sindelfingen, Germany).

### 2.5. Wettability Test

A drop shape analyzer (Attention Theta Life, Biolin Scientific, Espoo, Finland) was used to assess the wettability of the coatings. The wettability of the material was evaluated by the falling drop method using water (~4 µL) at room temperature (*n* = 5 for sample). The contact angle (CA) was analyzed for 10 s.

### 2.6. Corrosion Resistance

Corrosion resistance tests were carried out using a potentiostat/galvanostat (Atlas 0531; Atlas Sollich, Gdańsk, Poland) in a three-electrode configuration, with AtlasCorr05 software (Atlas 0531) employed for the calculation of corrosion parameters, including corrosion current density and corrosion potential. The working electrode consisted of a titanium alloy sample, the counter electrode was a platinum rod, and a saturated calomel electrode served as the reference. All electrodes were immersed in 200 mL of simulated body fluid (SBF) with the following composition [23,25]: NaCl—8 g/L; KCl—0.4 g/L; NaHCO_3_—0.35 g/L; CaCl_2_—0.14 g/L; Na_2_HPO_4_—0.06 g/L; KH_2_PO_4_—0.06 g/L; MgSO_4_—0.01 g/L; and glucose—1 g/L. A fresh portion of SBF was used for each sample, and the solution temperature was maintained at 37 °C throughout the tests.

Initially, the open circuit potential (OCP) was recorded. Potentiodynamic polarization curves were then measured, with the applied potential ranging from approximately −1.0 V to +1.0 V at a scan rate of 1 mV/s. The selection of this potential range was based on previous research [23,24].

### 2.7. Mechanical Studies

The nanomechanical properties of the samples (*n* = 10) were characterized using a nanoindenter (Alemnis AG, Thun, Switzerland) equipped with a Berkovich diamond indenter tip featuring a three-sided pyramidal geometry and an apex angle of 124.4°. Indentation tests were conducted under a maximum applied load of 20 mN, with both loading and unloading durations set to 0.5 s. Hardness (H), maximum indentation depth, and Young’s modulus (E) were determined based on load–displacement data analyzed using the Oliver–Pharr method [26]. To obtain the Young’s modulus from the reduced modulus, a Poisson’s ratio of 0.3 was assumed for the tested coatings.

The bending test was performed using a universal testing machine TestControl II 5 kN (ZwickRoell, Wrocław, Poland). The test was conducted in a three-point configuration, in which the specimen was supported on two symmetrically positioned supports spaced 30 mm apart. A preload force of 0.5 N was applied before the actual measurement. The crosshead speed during the bending test was set to 3.6 mm/min. The flexural strength was calculated using the mathematical relationship between the bending moment (*M*) and second moment of area (*W*), in accordance with Equation (1) [27]. The section modulus is defined as the ratio of the second moment of area and the distance from the neutral axis to the outermost fiber [28]. For a specimen with a circular cross-section, *W* takes the form described by Equation (2).

(1) $$\sigma =\frac{M}{W}$$

(2) $$W=\frac{\pi d^{3}}{32}$$

The bending moment can be calculated using Equation (3) [29], where *F* refers to the maximum applied load and *L* is the length of the support span.

(3) $$M=\frac{F\;L}{4}$$

## 3. Results and Discussion

### 3.1. Surface Examinations

Using an electric current applied to a solution of hydroxyapatite and chitosan, chains of chitosan and hydroxyapatite-derived compounds (CaCO_3_, Ca(OH)_2_) can be co-deposited. This process is accompanied by cathodic reduction of water and production of hydrogen gas at the cathode surface. The concentration of the solution components and the process parameters (time and voltage) influence the resulting mass and structural properties of the deposit. Therefore, by modifying them, it is possible to tailor the deposit’s properties to the needs of a given application [30].

**Figure 1:**
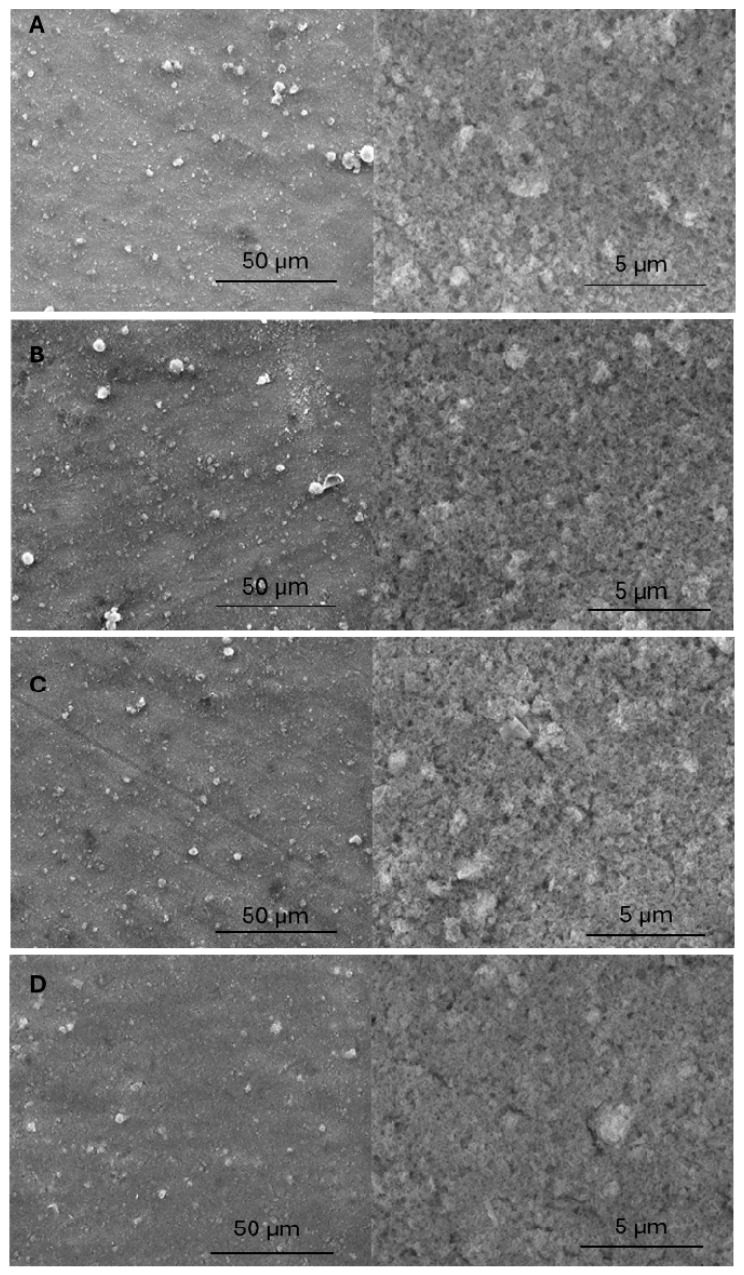
SEM images of composite coatings; (**A**) CS/nHAp10_2; (**B**) CS/nHAp10_5; (**C**) CS/nHAp20_2; (**D**) CS/nHAp20_5.

Based on the images obtained from the scanning electron microscope (Figure 1), it can be concluded that all obtained coatings were continuous, with no cracks observed. The size of the agglomerates decreased with increasing applied voltage. The influence of settling parameters on the formation of agglomerates was also confirmed by Bartmański M. [31]. His research indicates that extending the deposition time from 1 to 2 min increases the concentration of nHAp agglomerates on the coating surface [31]. The presence of agglomerates is also widely reported in the literature on chitosan–hydroxyapatite composite coatings, where their formation is suggested to result from electrostatic interactions and the tendency of HAp particles to aggregate within the polymer matrix. Studies on modifying the coating composition have shown that increasing the proportion of an inorganic phase, such as titanium oxide (TiO_2_), relative to HAp leads to a smoother and more uniform surface. It should also be emphasized that despite the improved surface morphology, the coatings described in this study exhibited a tendency to develop cracks within the coating [12].

**Figure 2:**
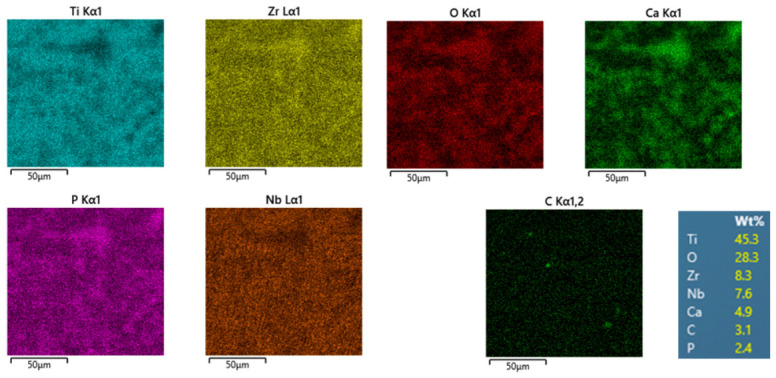
EDS map of element distribution on the surface (CS/nHAp10_5).

EDX analysis (Figure 2) confirms the successful deposition of CS and nHAp by the EPD method. Characteristic peaks in the spectrum corresponding to carbon, oxygen, calcium, and phosphorus are consistent with the expected presence of CS and nHAp, respectively. The EDS maps presented in Figure 2 also confirm the mostly uniform distribution of these elements. The presence of titanium, zirconium, and niobium is related to the presence of these elements in the substrate on which the coatings were deposited.

**Figure 3:**
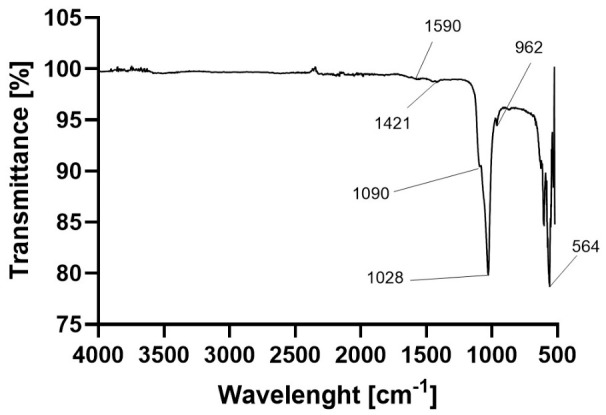
The FTIR spectra for chosen chitosan–hydroxyapatite coating (CS/nHAp20_5).

The FTIR analysis results of the selected coating are shown in Figure 3. The FTIR spectrum shows peaks around 564 and 963 cm^−1^, which are associated with the phosphate groups [12,13,32]. The peak observed at 1421 cm^−1^ is attributed to carbonate bonds [12]. However, according to Varma R. et al., the peaks at 1421 and 1486 cm^−1^ correspond to the C–H side chain bending of —CH_2_OH [33]. The peak around 1590 cm^−1^ is related to N-H bending vibrations in the amide group of chitosan [34]. The FTIR peak observed at 1090 cm^−1^ is characteristic of C–O bond stretching in polysaccharides [12,32]. The bands at 1028 cm^−1^ correspond to C-O stretching [20]. Based on these research results, it can be concluded that a composite containing both chitosan and hydroxyapatite was obtained.

**Figure 4:**
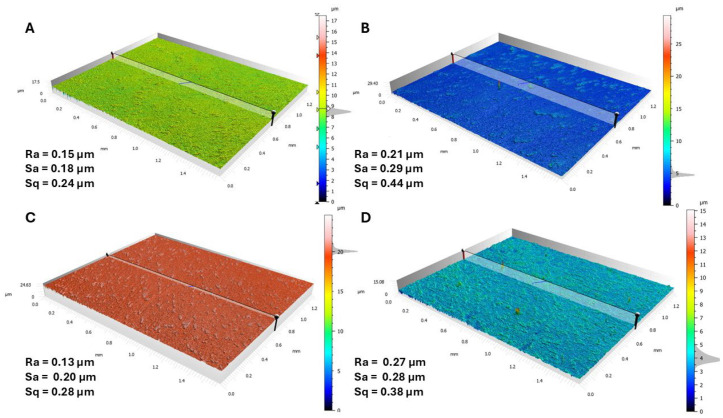
Surface topography of (**A**) CS/nHAp10_2; (**B**) CS/nHAp10_5; (**C**) CS/nHAp20_2; (**D**) CS/nHAp20_5.

The roughness of the surface of the composite coatings obtained was examined using a 3D optical profilometer in confocal microscopy. The measurement results are presented in Figure 4. Three characteristic parameters describing the surface topography were analyzed—linear parameter Ra (arithmetic mean height), as well as 3D surface area parameters, Sa (arithmetic mean height), and Sq (root mean square height). Based on the data obtained, a clear influence of both deposition time and voltage on the surface structure of the coatings can be observed. The lowest roughness values were observed for the CS/nHAp20_2 sample (Ra = 0.13 μm; Sa = 0.20 μm; Sq = 0.28 μm), suggesting that a shorter deposition time at a higher voltage promotes the formation of a more homogeneous and relatively smooth layer. In contrast, the highest roughness parameter values were observed for the CS/nHAp20_5 sample (Ra = 0.27 μm; Sa = 0.28 μm; Sq = 0.38 μm). This means that prolonging the process leads to increased deposition of the coating and the formation of more pronounced surface irregularities. When comparing the effect of voltage, it can be concluded that with a short deposition time (2 min), the use of a higher voltage (20 V) results in a smoother surface compared to deposition at 10 V. However, for a longer time (5 min), the effect of voltage is no longer clear—roughness increases in both cases, but the differences between 10 V and 20 V are less pronounced. The results obtained indicate that the key factor determining the roughness of the coating surface is the deposition time, while voltage plays a secondary role, mainly affecting the morphology of the coating in the initial stages of the process. Increasing the deposition time leads to more intensive material deposition and an increase in the Ra, Sa, and Sq parameters, which may be related to the formation of agglomerates of nHAp and chitosan particles in the coating matrix. The obtained results are comparable with the Sa values of nanohydroxyapatite coatings, which were deposited under the same conditions and had values around 200 nm [22]. In a study conducted by Ansari Z. et al. [28], where chitosan–hydroxyapatite composites (10, 50, and 60 wt.% HAp) were synthesized on Ti6Al4V alloy. The Ra values were more than ten times higher (ranging from 2.88 to 12.93 µm), although it should be noted that the coating thickness ranged between 15 and 50 µm. Surface roughness increased with higher HAp content, which was attributed to increased porosity and the presence of agglomerates [32].

Surface roughness influences biological effects. In the body, vascular cells constantly interact with tissues with nanostructured surface features due to the presence of proteins (such as collagen and elastin) embedded in the vessel wall. Studies have shown increased adhesion of endothelial and vascular smooth muscle cells to nanostructured materials [35]. In their research, K. Zhou et al. [36] developed surface gradients with a wide range of nanomicrometer roughness on a magnesium surface to investigate the responses of EC and SMC. Endothelial cells and smooth muscle cells interacted significantly differently with surface topographic patterns. That was particularly evident in the upper roughness range Sa between 0.7 and 2.0 μm. They identified the optimal range of surface microroughness Sa between 1.0 and 2.0 μm, which most significantly increases the selectivity of endothelial cell adhesion over smooth muscle cells [36]. However, increasing surface roughness may lead to greater platelet adhesion because additional surface roughness typically means a larger area exposed to platelets. Materials with micrometer-scale topography have been shown to exhibit greater platelet adhesion during the initial period of contact with blood (up to 5 min) [37].

The Sa parameter of the obtained materials ranged from 0.18 to 0.29 µm. According to the literature, this range of surface roughness is below the level of selective stimulation. This suggests that proliferation of both endothelial and smooth muscle cells is expected on the obtained coatings. Since a surface with Sa in the range of 0.18–0.29 µm is relatively smooth, it can be concluded that such a material is not expected to promote platelet adhesion. Rather, reduced or moderate platelet adhesion is anticipated, which also depends on other surface properties such as wettability and surface energy. Limiting smooth muscle proliferation is crucial for preventing restenosis; therefore, supporting this function through the release of an appropriate drug/active substance will be required. Although the influence of micro-nanostructures on cell proliferation, adhesion, and other behaviors is not as strong as the influence of drugs, they possess unique advantages in directing and regulating cell distribution and orientation [38].

**Figure 5:**
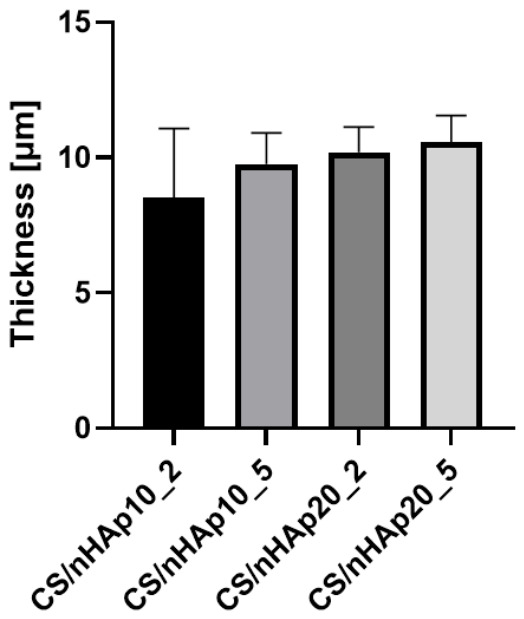
The thickness of the coatings. Values are shown as mean ± SD; *n* = 10.

The coating thickness was evaluated using a DualScope coating thickness gauge. As shown in Figure 5, the measured thickness ranged from 8.5 to 10.6 µm. The increase in both deposition time and voltage is associated with an increase in coating thickness, which is consistent with studies reported in the literature [39].

### 3.2. Wettability Test

Wettability is important for determining the potential for biological integration. Surface wettability influences cell and protein adhesion [24], which are fundamental for implant–tissue integration, as well as healing and regeneration processes. There are reports indicating that cell adhesion depends primarily on surface wettability, but it is also influenced by the type of surface functional groups, their surface density, and the specific cell type [40]. Additionally, hydrophilic surfaces better bind water and components of body fluids, which facilitates cell migration, proliferation, and differentiation. Another important aspect is infection control: hydrophilic surfaces tend to promote bacterial adhesion, whereas hydrophobic coatings help limit it [41]. Although hydrophilic implant surfaces are highly desirable and designed to improve biocompatibility, they can negatively affect hemocompatibility. In materials intended for blood contact, hydrophilic surfaces may promote clot formation. In this case, superhydrophobic materials are more effective, as they can reduce platelet adhesion and exhibit anti-hemolytic properties [42,43].

**Figure 6:**
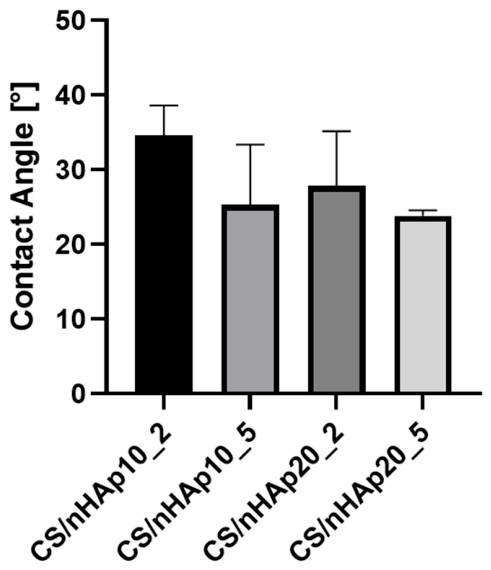
Wettability test results. Values are shown as mean ± SD; *n* = 5.

Figure 6 presents the results of surface wettability tests for composite coatings. A change in wettability was observed depending on the deposition parameters used. The contact angle value decreased both with increasing deposition time and with the application of higher voltage. The average contact angle values for the fabricated composite coatings ranged between 24° and 35°. The lowest value was recorded for CS/nHAp20_5 (24 ± 1°), while the highest was observed for CS/nHAp10_2 (35 ± 4°). According to the literature, increasing the hydroxyapatite content in the composite coating leads to a decrease in the contact angle [32]. Chitosan typically exhibits a contact angle of around 70–80° [8], while hydroxyapatite ranges between 20° and 40° [22]. In previous studies [22], using the same deposition parameters for nHAp, the contact angle values were above 30° in all cases except for the coating deposited at 20 V for 5 min, where the contact angle dropped to 22°. These results indicate that the wettability of composite materials is similar to that of pure nanohydroxyapatite coating, which may be attributed to the predominance of nHAp relative to CS. In the electrolyte, the content of nHAp was ~73.7%, and CS ~26.3%. Additionally, the results of EDX analysis (Figure 2) confirmed increased calcium content on the surface of the coatings.

### 3.3. Corrosion Resistance

**Figure 7:**
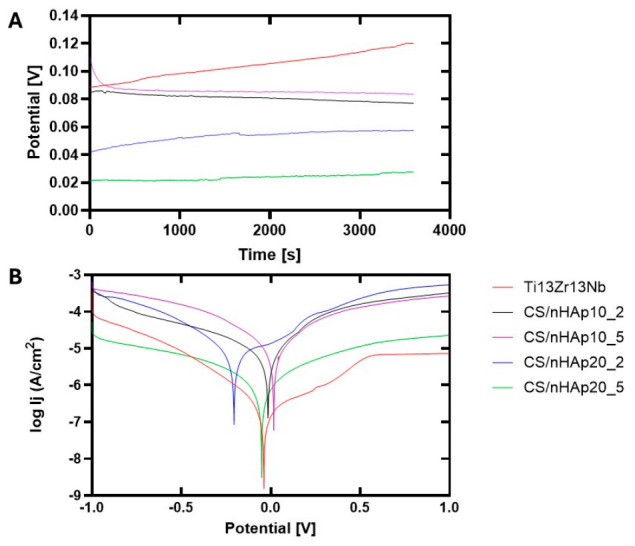
Corrosion behavior of uncoated Ti13Zr13Nb alloy and chitosan–nanohydroxyapatite composites; (**A**) open circuit potential vs. time; (**B**) potentiodynamic corrosion curves.

**Figure 8:**
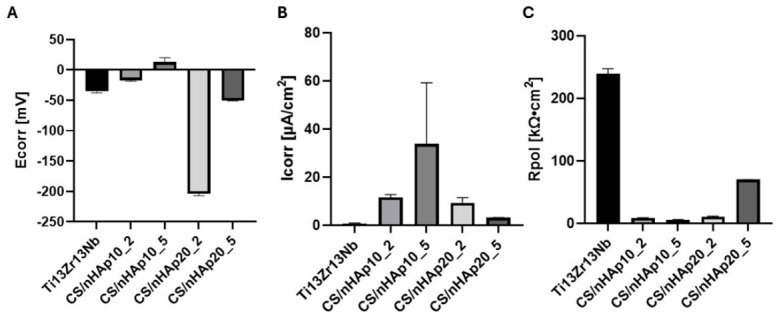
Results of corrosion tests: (**A**) Corrosion potential (Ecorr); (**B**) Corrosion Current Density (Icorr); (**C**) Polarization Resistance (Rpol). Values are shown as mean ± SD; *n* = 3.

The results of corrosion tests are presented in Figure 7 and Figure 8. The open circuit potential (OCP) versus time was measured for each sample in SBF solution at 37 °C. After one hour of observation, all tested samples showed stabilization of the OCP values. The coatings showed OCP stabilization in the range from 0.02 V to about 0.09 V, while the reference Ti13Zr13Nb sample stabilized around 0.12 V. Higher OCP values were observed for coatings applied at lower voltages. No significant influence of deposition time on OCP was observed. The OCP value for CS/nHAp10_5 is significantly higher than other composites, but the highest value is given to the titanium alloy. It should also be noted that the OCP values differ from the corrosion potential evaluated using the AtlasCorr05 software via Tafel extrapolation. In the conducted studies, the OCP value is not considered a reliable indicator of corrosion tendency; this approach is reflected in the literature [44].

Sorkhi L. et al. [12] confirmed the improvement in corrosion properties after the use of composite coatings based on chitosan and hydroxyapatite. Additionally, the improvement in corrosion resistance was influenced by the use of titania particles which can fill the gaps between HAp particles [12]. A more positive corrosion potential usually indicates better corrosion resistance. Based on this, it can be concluded that composites deposited at 10 V, regardless of deposition time, enhance corrosion resistance compared to pure titanium alloy. Coatings deposited at higher voltage values exhibited greater thickness, but an increased surface heterogeneity was also observed. This may have contributed to the formation of so-called corrosion channels, reducing corrosion resistance. These channels can act as pathways for ion diffusion, weakening the protective function of the coating. The increased porosity and microstructural defects resulting from the rapid movement and deposition of particles at high voltage contribute to the formation of heterogeneities, which in turn limit corrosion resistance [16]. Studies of the titanium dioxide layer formed on the titanium surface have shown that it can provide increased corrosion resistance only when it is continuous across the entire alloy surface. If the layer is characterized by cracks and discontinuities, so-called “corrosion channels” form, accelerating material degradation [2].

The Icorr and Rpol parameters indicate how quickly corrosion begins. Considering the results presented in Figure 8, low Rpol and high Icorr indicate active corrosion and poor protection. Especially in the case of CS/nHAp10_5, Ł. Pawłowski et al. [16] stated that the increase in Icorr for the coatings may be associated with their increased heterogeneity [16]. The CS/nHAp10_5 coating was characterized by a large number of agglomerates on the surface. Hydroxyapatite coatings are heat-treated to improve their properties. The proposed CS/nHAp composite was not heat-treated, which may result in lower corrosion resistance compared to sintered coatings. After heat treatment, the coating is compact and well-crystallized with dense and uniform particle distribution, which provides a barrier to the corrosion mechanism [45].

Coating CS/nHAp20_5 exhibits a moderate Ecorr, which means that the surface is neither highly corrosion-resistant nor highly active. Its Ecorr is also only slightly more negative than that of pure metal. However, among all the composite coatings, it shows the lowest Icorr and the highest Rpol indicating strong resistance to corrosion current flow, which also suggests an effective protective barrier.

### 3.4. Mechanical Studies

Nanoindentation tests showed the influence of electrophoretic deposition parameters on hardness (H) and reduced Young’s modulus (E) (Table 2). The mechanical properties of CS/HAp composites may be influenced by several factors, such as: the size of HAp particles, the mechanical strength of the chitosan matrix, the interactions at the interface between CS and HAp, and the good distribution of HAp in the CS matrix [46]. Coatings deposited at a voltage of 10 V (CS/nHAp10_2 and CS/nHAp10_5) were significantly harder and stiffer than those obtained at 20 V. The CS/nHAp10_2 coating had the highest hardness and Young’s modulus, indicating the greatest resistance to permanent plastic and elastic deformation. CS/nHAp10_5 also showed high hardness and Young’s modulus, although slightly lower than CS/nHAp10_2. The lowest values were obtained for coatings deposited at 20 V, especially CS/nHAp20_2. The CS/nHAp20_5 coating was characterized by medium hardness and the lowest Young’s modulus, which means the highest elasticity among all coatings. Surface morphology also affects hardness and stiffness. Large agglomerates cause local structural heterogeneity, and agglomerates are also associated with surface defects and reduced corrosion resistance, as confirmed in the case of CS/nHAp10_5. In contrast, CS/nHAp20_5 was characterized by the greatest structural homogeneity, smaller agglomerates and their uniform distribution, which facilitated consistent and repeatable measurement results and ensured the best corrosion resistance.

The observed differences in the mechanical properties of the electrophoretically deposited chitosan–nanohydroxyapatite coatings can be attributed to the influence of deposition parameters on the microstructure and phase distribution within the coating. Based on the experimental results, it can be assumed that parameters such as deposition voltage and process time influenced particle mobility, deposition rate, and hydrogen evolution intensity during the EPD process. This consequently influenced the coating’s uniformity and continuity, as well as its hardness and Young’s modulus. Additionally, the distribution and interaction of the chitosan matrix and hydroxyapatite particles may have had a significant impact.

The results of mechanical tests confirm the observations known from the literature that bioactive composite coatings should combine appropriate stiffness with simultaneous flexibility to reduce the stress shielding effect and improve integration with bone tissue [47]. In this context, composites deposited at 20 V, despite lower hardness and Young’s modulus values, exhibit parameters closer to cortical bone (10–30 GPa), which may be beneficial for long-term implant stability [48]. The appropriate mechanical properties of the HAp/CS composite scaffold can be attributed to its hybrid nanostructure, which combines the hardness of HA crystals with the good tensile properties of chitosan fibres [49]. Surface modification in the form of a combination of chitosan and hydroxyapatite significantly reduces Young’s modulus, which is approximately 79 GPa [50] for Ti13Zr13Nb. The significantly higher stiffness of the implant causes the load to be transferred mainly by the implant, which protects the bone from physiological load and leads to bone resorption. This effect, known as ‘stress shielding’, is the main cause of complications such as aseptic loosening of the implant or periprosthetic fractures [51,52,53].

In the case of stents, their biomechanical interaction with the target blood vessels is a critical factor influencing long-term clinical outcomes. Unfortunately, excessive mechanical stress can trigger adverse arterial responses, and such damage has been linked to the development of restenosis. Stents with lower stiffness are better able to conform to the tapering geometry of the artery, leading to reduced arterial wall stress [54]. Excessive coating hardness may increase the risk of tissue damage, trigger inflammation, and even contribute to the development of restenosis. On the other hand, insufficient coating hardness may lead to faster wear and degradation of the coating. Coatings characterized by lower elastic modulus are generally softer and more compliant, enabling improved conformity to the vessel wall and potentially reducing the risk of endothelial injury. For optimal performance, the coating must be capable of withstanding mechanical deformation during stent deployment without cracking, as excessive stiffness may compromise structural integrity under physiological loading conditions.

**Figure 9:**
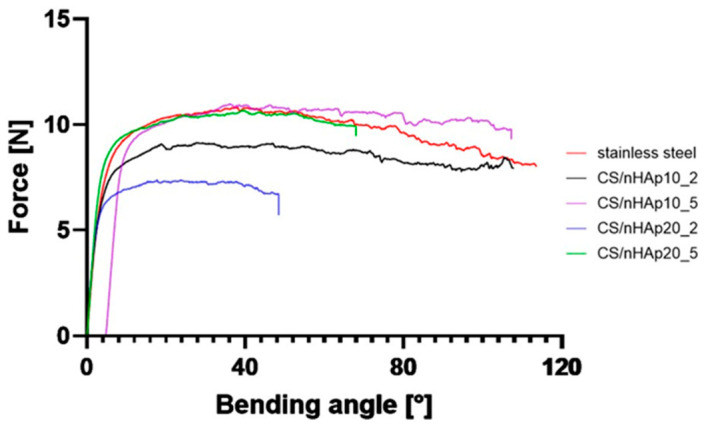
Bending angle values under standard force for uncoated and composite-coated stainless steel wires.

In order to evaluate the bending behavior of the materials, a three-point bending test was conducted. This widely used method in biomaterial research involves positioning the specimen horizontally on two supports and applying a vertical load at the midpoint until deformation or failure occurs [55,56,57]. Load and displacement data were recorded continuously to generate a force versus deflection curve. Table 2 presents the flexural strength values for the stainless steel wire and those coated with composite CS/nHAp coatings. The flexural strength values were calculated according to Formulas (1)–(3). For an object with a circular cross-section and a diameter of 1 mm, the second moment of area (W) was assumed to be 0.10 mm^3^. Figure 9 shows a graph of the bending angle as a function of the applied force. The chitosan/nHAp coatings were not heat-treated, and their thickness is less than 11 µm; therefore, they should not significantly affect the flexural strength of the steel wire. In the case of ceramic layers, heat treatment is necessary to create a chemical bond between the coating and the metallic substrate. Temperature also causes sintering of the ceramic particles, affecting their thermal expansion, and shrinkage of the ceramic layer induces stresses in the material. Furthermore, deformation causes more significant stresses and cracks to develop at the boundaries of large agglomerates, which can result in delamination and chipping of these parts of the ceramic layer [58]. The coating serves a protective and bioactive function rather than reinforcing the metal substrate. Cracking or chipping of the coating during bending may have only a minimal impact on flexural strength, and only if microcracks were to initiate in the substrate. Therefore, changes of less than 2% can be considered negligible—CS/nHAp20_5 (1.21%), CS/nHAp10_5 (1.67%). A decrease in flexural strength was observed for CS/nHAp20_5 (1.15%), CS/nHAp10_2 (15.43%), and most notably for CS/nHAp20_2, which exhibited a reduction of 31.76%.

### 3.5. Analysis of Surface Response to Wire Deformation

**Figure 10:**
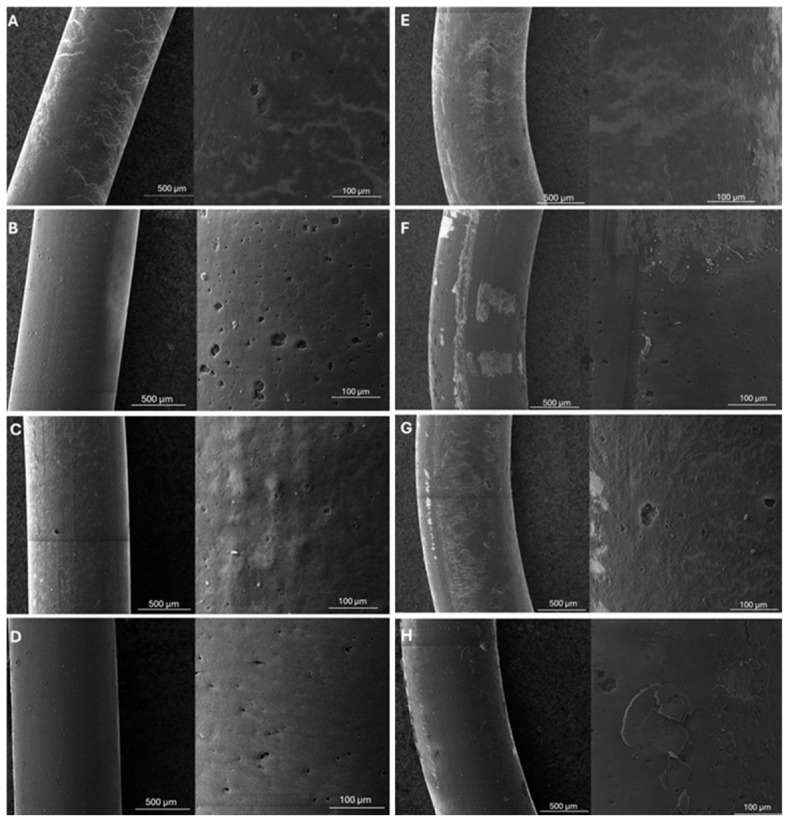
SEM images of composite coatings before wire deformation (**A**–**D**) and after wire bending (**E**–**H**); (**A**,**E**) CS/nHAp10_2; (**B**,**F**) CS/nHAp10_5; (**C**,**G**) CS/nHAp20_2; (**D**,**H**) CS/nHAp20_5. Magnification: 100× and 500×.

The aim of the study is to determine the behavior of the coating under wire deformation. For this purpose, SEM imaging of the wire surfaces was performed in the central region of the wire and at the locations of bending (Figure 10). It can be observed that unlike the coatings deposited on cylindrical titanium alloy samples, those deposited on steel wires exhibit a rough structure. The increase in deposition time correlated with the formation of surfaces characterized by local discontinuities. At 20 V and 5 min, crack propagation originating from isolated microvoids was observed. The CS/nHAp20_2 coating exhibits small surface depressions as well as surface irregularities, which may suggest the formation of gas bubbles beneath the coating during the deposition process. The coating deposited under the lowest parameter settings displays dendritic streaks, although these do not indicate damage or cracking of the coating.

After wire bending, the least damage was observed in the CS/nHAp10_2 coating, where damage was limited to the point of applied pressure. The most severe damage occurred in the CS/nHAp10_5 coating, where wire deformation led to extensive cracking and exposure of the metallic substrate. The CS/nHAp20_2 coating largely retained its structural integrity, with only minor material loss observed on the outer side of the wire at the bend. In the case of the coating deposited using the highest parameter settings, chipping and propagation of pre-existing cracks were observed; however, no exposure of the metallic substrate was detected.

The modulus of elasticity of stainless steel is approximately 200 GPa [59,60], while the modulus of elasticity of the resulting coatings is significantly lower. Mechanical mismatch leads to shear forces at the metal–coating interface and microcracks. If the coating does not exhibit sufficient adhesion to the substrate, delamination of the coating or even detachment of material fragments during deformation may occur. Coatings characterized by high hardness and stiffness are typically accompanied by brittle deformation. Consequently, cracks easily form, and their subsequent rapid propagation can lead to premature failure of the coating and, consequently, the object itself [61]. Decreasing the stiffness of the coating lowers the risk of delamination and buckling when the coating is free of defects. However, a reduction in coating stiffness will not promote breaking of web defects during deformation [62]. Additionally, coating thickness may also influence the observed effect after deformation. Studies on coatings produced by electroplating chromium have shown that the density of cracks before and after loading depends on coating thickness and increases as the coating becomes thinner. Pre-existing cracks located at the substrate–coating interface also play a significant role [63].

The obtained coatings do not have a reinforcing function and should not significantly affect the strength properties of the metal. Under the influence of applied load and material deformation, the CS/nHAp10_2 coating demonstrated flexibility and good adhesion to the substrate. The sample with the CS/nHAp10_5 coating, which exhibited the highest flexural strength, also showed the most severe damage, including cracking and exposure of the metal substrate. Although the overall strength of the system was satisfactory, the coating most likely did not exhibit adequate adhesion to the substrate and was locally brittle. These properties often cause extensive damage. Despite the significant reduction in flexural strength observed for the CS/nHAp20_2 sample, no major coating damage was observed. The coating was thicker, characterized by lower hardness and a lower modulus of elasticity, which is associated with the material’s greater flexibility. Surface defects did not become sites of crack propagation during material deformation. Surface discontinuities on the side opposite the device’s pressure point may result from high stresses in these areas and higher shear forces at the metal–coating interface. The wire with the CS/nHAp20_5 coating showed a negligible decrease in flexural strength. However, the longer deposition time caused structural defects and most likely higher internal stresses. Existing micropores became sites for crack initiation. However, despite partial damage, the metal substrate was not exposed. This means that the coating still partially fulfilled its protective function. In tests conducted on titanium alloys, this coating demonstrated the greatest thickness of all tested variants. According to literature data, stresses within the layer vary with the level of elastic mismatch and coating thickness [64]. A higher coating thickness may result in greater brittleness [65]. This greater thickness could also be the reason why, despite cracking and chipping of coating fragments, the substrate was not exposed. Moreover, the material was not heat-treated after deposition (which is common practice for pure nanohydroxyapatite coatings [22,66]), and its adhesive properties may have been reduced when a thicker layer was applied.

## 4. Conclusions

Composite coatings of chitosan and nanohydroxyapatite were successfully deposited on titanium alloy substrates (Ti13Zr13Nb) using the electrophoretic deposition method at 10 V and 20 V for 2 and 5 min. EDX and FTIR analysis confirmed the presence of both chitosan and hydroxyapatite components. All obtained coatings were continuous, crack-free, and exhibited hydrophilic properties, with contact angles ranging from 24° to 35°. Both the roughness of the coatings at the level of several hundred nm and the hydrophilicity of the surface should support the adhesion of endothelial cells, which promotes endothelialization. The thickness of the coatings ranged from 8.5 to 10.6 µm. Corrosion studies showed that coatings deposited at 10 V improved corrosion potential, whereas those deposited at 20 V demonstrated lower corrosion current density and higher polarization resistance, indicating better corrosion protection. Mechanical testing revealed that coatings obtained at 10 V were harder and stiffer, while those deposited at 20 V, particularly CS/nHAp20_5, exhibited higher elasticity and lower Young’s modulus, suggesting better flexibility. Additionally, the bending strength was determined and the effect of deformation on the coating’s behavior was assessed using a three-point bending test. For this purpose, the coatings were mounted on 1 mm stainless steel wires. Although a slight decrease in flexural strength was observed for the CS/nHAp20_5 coating and local strain cracking occurred, the coatings still provided partial protection to the substrate, as evidenced by local spalling without full metal exposure. Overall, the results suggest that deposition parameters have a significant impact on the structural, mechanical, and protective properties of CS/nHAp coatings. Considering corrosion resistance and mechanical properties, the CS/nHAp20_5 coating demonstrated the lowest corrosion current density and the highest polarization resistance. Furthermore, this coating had the lowest stiffness and modulus of elasticity, suggesting the best flexibility. After deformation of the CS/HAp20_5 wire, despite the occurrence of local cracks, the substrate was not exposed.

## Figures and Tables

**Table 1:** Sample names and deposition parameters.

Sample Names	Parameters
CS/nHAp10_2	10 V, 2 min
CS/nHAp10_5	10 V, 5 min
CS/nHAp20_2	20 V, 2 min
CS/nHAp20_5	20 V, 5 min

**Table 2:** Results of mechanical studies. Values are shown as mean ± SD; *n* = 10.

Sample	Nanoindentation Test		Three-Point Bending Test	
	Hardness [GPa]	Reduced Young’s Modulus [GPa]	Maximum Load Force [N]	Flexural Strength sfM [MPa]
CS/nHAp10_2	0.89 ± 0.50	55.3 ± 0.79	9.14	685.50
CS/nHAp10_5	0.73 ± 0.09	41.8 ± 1.0	10.98	823.50
CS/nHAp20_2	0.12 ± 0.09	18.5 ± 0.1	7.37	552.75
CS/nHAp20_5	0.33 ± 0.06	12.7 ± 1.4	10.68	801.00
SS 316L	-	-	10.80	810.00

## Data Availability

The original contributions presented in this study are included in the article. Further inquiries can be directed to the corresponding authors.
